# Protective Effect of Anthocyanin from *Lonicera Caerulea* var. Edulis on Radiation-Induced Damage in Mice

**DOI:** 10.3390/ijms130911773

**Published:** 2012-09-18

**Authors:** Haitian Zhao, Zhenyu Wang, Fengming Ma, Xin Yang, Cuilin Cheng, Lei Yao

**Affiliations:** 1 School of Food Science and Engineering, Harbin Institute of Technology, Harbin 150090, China; E-Mails: zhaoht9999@163.com (H.Z.); mfm88492800@163.com (F.M.); yangxin940@163.com (X.Y.); luluyao2012@gmail.com (C.C.); yaoleiyl2000@163.com (L.Y.); 2 School of Forestry, Northeast Forestry University, Harbin 150040, China; 3 National Research Center of Soybean Engineering and Technology, Northeast Agriculture University, Harbin 150030, China

**Keywords:** anthocyanin, *Lonicera caerulea* var. edulis, ^60^Coγ radiation, mice, radioprotection

## Abstract

The radioprotective effect of anthocyanin extracted from *Lonicera caerulea* var. edulis (ALC), was studied in ICR mice. Different doses of ALC were intragastrically administered to mice once a day, prior to radiation. After two weeks, the mice received a one-time 5 Gy whole body ^60^Coγ radiation. The spleen index, thymus index, activities of superoxide dismutase (SOD) and glutathione peroxidase (GSH-Px), malondialdehyde (MDA) content, and glutathione (GSH) content in liver tissue were measured. Compared with the radiation control group, the levels of MDA in all ALC treated groups decreased significantly (*p* < 0.05). Moreover, the GSH content, activities of SOD and GSH-Px in liver tissue were enhanced significantly (*p* < 0.05) in all ALC groups. These results demonstrate that ALC may be a potential radioprotector, and a further study of the molecular mechanism is needed for further application.

## 1. Introduction

With the rapid development of nuclear technology in industry, agriculture, medicine, and other fields, the chances of radiation exposure and the possibility of radiation damage have increased. One study shows that the cellular damage induced by ionizing radiation is predominantly mediated through the generation of reactive oxygen species (ROS) [1]. These ROS react with proteins, nucleic acids, lipids, and other macromolecules, resulting in DNA strand breakage, DNA and protein cross-linking and lipid peroxide production [2]. Consequently, the metabolism, structure and function of cells, tissues and organs will be damaged, ultimately causing body injury. Some radioprotectors of both synthetic and natural products, e.g., antioxidants [3], sulfhydryl compound, estrogens, cytokines and growth factors [4] have been investigated in both *in vitro* and *in vivo* models to mitigate injuries caused by ionizing radiation. However, many of them had severe side effects, such as nausea, vomiting, hypotension, nephro- and neuro-toxicity [5]. Therefore, the search for new radioprotectors that are less toxic than the currently available compounds has drawn more and more attention in recent years.

*Lonicera caerulea* (common name Blue Honeysuckle) belongs to Caprifoliaceae family, *Lonicera* genus, widespread in Siberia, northeastern Asia, and Japan [6]. It has become one of the newly developing small berry fruit crops because of its high nutrional value and plentiful wild resource. The fruit is rich in anthocyanins, flavonoids and low molecular-weight phenolic acids, especially anthocyanins [7]. Researchers have reported activities of anthocyanins extracted from *Lonicera caerulea* (ALC) on antioxidation [8], ultraviolet radiation protection [6,9] antiproliferation [10] and anti-inflammation [11]. However, reports about the protective effects of ALC on ionizing radiation are scarce. In our previous work, we demonstrated that ALC was found to show remarkable scavenging activity on 2,2-Diphenyl-1-picrylhydrazyl (DPPH) radical, 2,2′-azinobis-(3-ethylbenzothiazoline-6- sulfonic acid) (ABTS) radical, hydroxyl radical (·OH) and superoxide anion radical (·O^2−^) [12]. Since whole body irradiation in mice can generate damaging ROS, compounds with ROS-scavenging characteristics may help to protect the body against irradiation-induced damage.

The purpose of the present study was to investigate the protective effect of anthocyanin extracted from *Lonicera caerulea* var. edulis (indigenous to the Greater Higgnan Mountains in northeast China) against ^60^Coγ radiation in mice. We focused on determining ALC effects on immune organ indices and liver antioxidant status.

## 2. Results and Discussion

### 2.1. Effect of ALC Treatment on Body Weight Change of Mice

**Figure 1:**
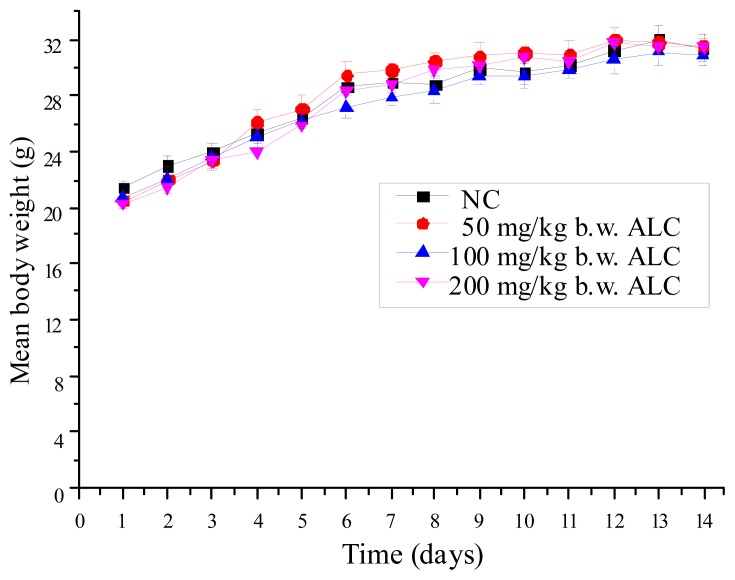
Effect of ALC treatment on body weight change of mice; the ALC-1/2/3 groups were given ALC daily by intragastric administration based on dose of 50, 100, and 200 mg/kg body weight (b.w.) per day for 14 consecutive days.

All groups of mice increased body weight gradually with the time of feeding (Figure 1) and there were no significant differences in ALC treatment groups with normal control group (NC group).

### 2.2. Effect of ALC on the Immune Organ Indices of Mice

Effect of ALC on the immune organ indices of mice are shown in Table 1. Our previous work has shown that, compared to the normal control, mice treatment with ALC alone (in dose of 50–200 mg/kg body weight), did not show any significant difference in spleen index or thymus index (data not shown). The influence of 5Gy ^60^Co γ-ray whole body irradiation on weight loss and atrophy in immune organs of mice was investigated. The absolute weights of spleen and thymus in the radiation control (RC) group were 44.18 ± 3.94 and 21.65 ± 2.59 mg, respectively, and significantly (*p* < 0.05) lower than levels in the NC group (153.62 ± 8.68 and 76.65 ± 6.76 mg). Sleep weight and thymus weight in ALC 100 and 200 mg/kg body weight groups are significantly higher than in the RC group (*p* < 0.05). This trend was also found in data of the immune organ indices. The thymus index and spleen index of mice in radiation control group (RC) were significantly lower than the NC group (*p* < 0.05). The thymus index and spleen index of the middle and high doses of ALC (100 and 200 mg/kg body weight) groups were significantly higher than the radiation control group (*p* < 0.05), but lower than the NC group (*p* < 0.05).

The immune system is very sensitive to ionizing radiation. The local damage to immune cells, tissues, and organs will inevitably induce damage to other systems, resulting in organism infection, bleeding, and various diseases. Radiation can cause serious damage to the immune organs of mice. Our data demonstrate that ALC treatment significantly reduces the trend of atrophy of the spleen and thymus in irradiated mice. However, ALC is not able to totally reverse the effect of radiation on spleen and thymus indices.

### 2.3. Effect of ALC on Oxidant/Antioxidant Status in Liver Tissue

**Figure 2:**
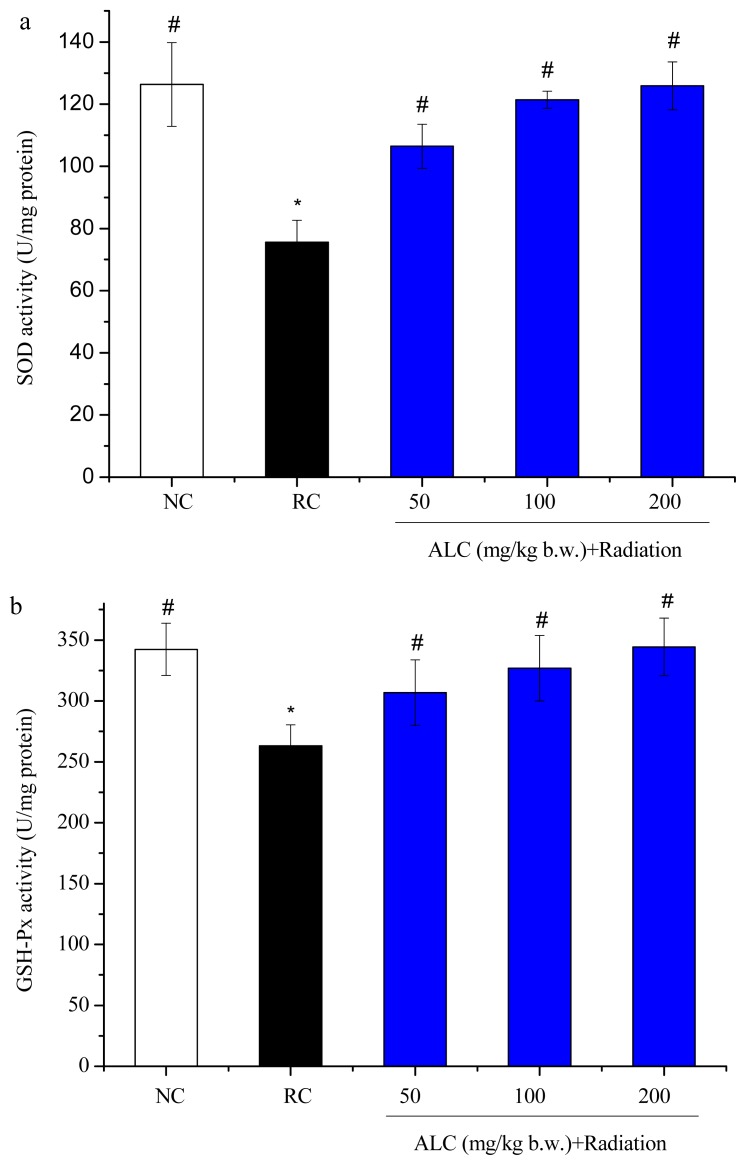

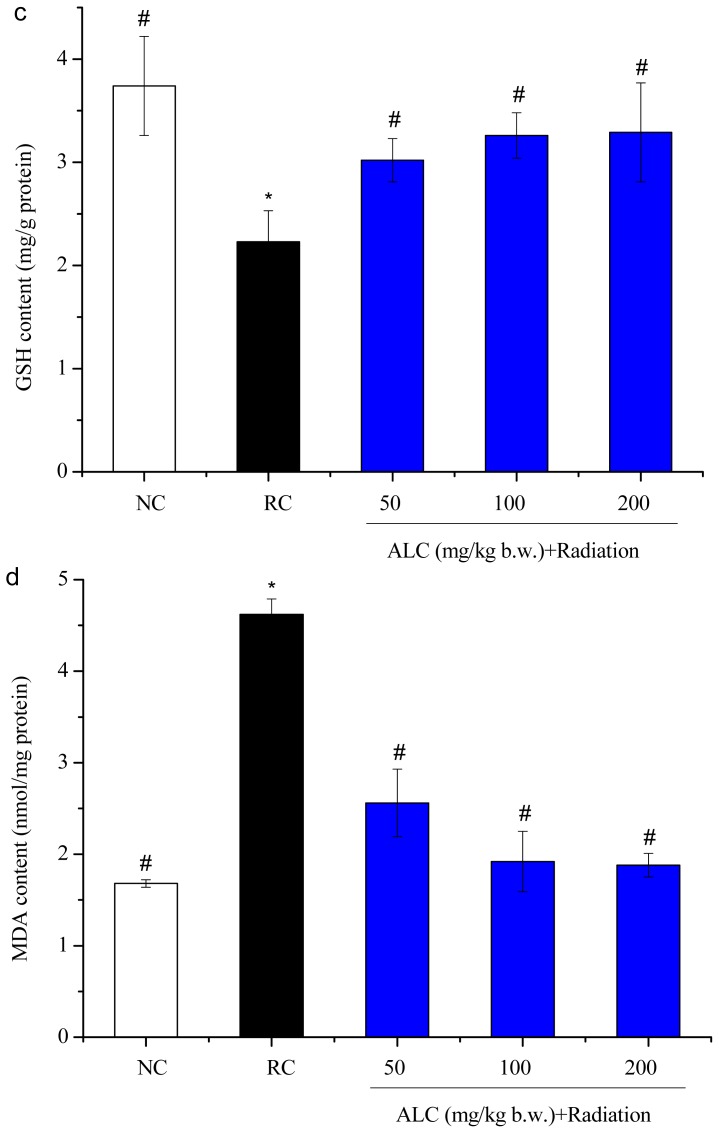
Effect of ALC on SOD activity (**a**), GSH-Px activity (**b**), GSH content (**c**) and MDA content (**d**) in liver tissue; Values= means ± SD, (*n* = 10) * *p* < 0.05 *vs*. NC group; ^#^ *p* < 0.05 *vs*. RC group.

Effect of ALC on oxidant/antioxidant status in liver tissue of mice after ^60^Coγ radiation is shown in Figure 2. Compared with the radiation control, administration of ALC significantly increased activities of superoxide dismutase (SOD) and glutathione peroxidase (GSH-Px) in liver tissue (*p* < 0.05) in a dose-dependent manner. The malondialdehyde (MDA) levels in liver tissue were significantly decreased in all of the ALC-treated groups, compared with the radiation control group (*p* < 0.05). The glutathione (GSH) content also effectively increased in all ALC treated groups (*p* < 0.05); there was a trend toward improvement with ALC dose.

Ionizing radiation causes the body to produce large amounts of ROS. Imbalance between production of ROS and antioxidant defense can result in oxidative stress [13], and the ensuing tissue damage may be involved in certain disease processes. Radiation injury is therefore influenced by the cellular antioxidant status [14]. Some reports indicate that several natural antioxidant compounds from plants exhibit radioprotective properties, such as polysaccharides [15,16], alkaloids [1] and polyphenols [17,18]. In our previous work, we demonstrated that ALC was found to show remarkable antioxidant activity *in vitro* [12]. Other authors have also proved anthocyanins to be good antioxidants in other models *in vitro* and *in vivo* [19,20].

MDA, an end-product in the oxidation of polyunsaturated fatty acids, is considered a useful indicator of lipid peroxidation [21]. Due to its high cytotoxicity and inhibitory action on protective enzymes, it has been suggested that MDA itself acts as a tumor promoter and a co-carcinogenic agent [22]. Hence, the reduction of radiation-induced MDA is desirable [23]. Under normal conditions, the inherent defense system, including GSH and the antioxidant enzymes, protects against oxidative damage [24]. Superoxide dismutases (SODs) belong to a ubiquitous family of enzymes that function to efficiently catalyze the dismutation of superoxide anions [25]. GSH-Px is a selenoprotein which reduces lipidic or nonlipidic hydroperoxides, as well as H_2_O_2_ while oxidizing GSH [26].

The decrease in GSH content, SOD and GSH-Px activities and the increase in MDA content in liver tissues post-irradiation as recorded in the present study are in agreement with those of previous studies [27–29]. This could be due to an enhanced utilization of the antioxidant system as an attempt to detoxify the free radicals generated by radiation [30]. We observed that pre-administration of ALC could potently inhibit oxidant damage induced by γ-radiation. The ingestion of ALC can remarkably enhance GSH content, SOD and GSH-Px activities. The MDA content in ALC treatment groups was also highly decreased (Figure 2). The increased activity of antioxidant enzymes reduce oxidative damage in ALC-treated irradiated mice, and may be attributed to facilitating the replacement of lost antioxidase activity in irradiated tissue or free radical scavenging effects of ALC.

## 3. Experimental Section

### 3.1. Chemicals and Reagents

The procedure for preparing ALC in laboratory was described previously [12]. In brief, The berry fruits were extracted with an aqueous solution of ethanol/water/hydrochloric acid (60:40:0.1, *v*/*v*/*v*) on an ultrasonic apparatus (KQ-500DB, Kunshan Ultrasonic Instruments Co., Ltd., Kunshan, China) at room temperature for 1 h. The primary extract obtained was purified on a column packed with X-5 macroporous resin (Nankai Hecheng Science & Technology Co., Ltd, Tianjin, China). The column was washed with deionized water, and subsequently the absorbed anthocyanins were eluted with 60% ethanol. The ethanol extract was then concentrated by vacuum evaporation and the anthocyanin powder was obtained by freeze-drying. The profile of ALC is as follows: cyanidin-3,5-diglucoside (3.81%), cyanidin-3-glucoside (74.28%), cyanidin 3-rutinoside (9.87%), pelargonidin-3-glucoside (2.36%), peonidin 3-glucoside (7.28%), peonidin 3-rutinoside (2.40%). The total anthocyanin content is 64.8%. The SOD, GSH-Px, GSH and MDA assay kits were purchased from Nanjing Jiancheng Bioengineering Institute, Nanjing, China.

### 3.2. Animals

Male ICR mice, approximately 7–8 weeks old and weighing 20 ± 2 g, were obtained from Harbin Veterinary Research Institute, Chinese Academy of Agricultural Science (CAAS, license number: SCXK (HEI) 2006-009). All animal experiments were performed under approval of the local Institutional Animal Care and Use Committee (IACUC).

### 3.3. Irradiation

Whole-body gamma-irradiation was performed at the Institute of Application of Atomic Energy, Heilongjiang Academy of Agricultural Sciences, Harbin, China. Animals were irradiated at an acute single dose level of 5 Gy (below the sublethal dose on mice) delivered at 95 cm source-to-skin distance (SSD). The dose rate was 1.0 Gy/min.

### 3.4. Experimental Design

Fifty ICR mice were randomly divided into five groups of 10 animals each, namely, normal control group (NC group), radiation control group (RC group), ALC treatment + radiation group (ALC1, ALC2 and ALC3group). Mice in the ALC-1/2/3 groups were given a daily intragastric administration based at doses of 50, 100, and 200 mg/kg body weight per day for 14 consecutive days. The NC group and NC group were administered with double distilled water. Body weight changes of the mice in all groups were monitored every 24 h. After continuous administration for two weeks, all mice, except the NC group, were exposed to ^60^Coγ-ray for whole-body radiation. Mice were sacrificed by cervical dislocation, 24 h after radiation. Tissues were removed immediately and frozen with liquid nitrogen and stored at −80 °C.

### 3.5. Thymus and Spleen Indices

All mice were weighed and then sacrificed by cervical dislocation. Spleen and thymus were excised and weighed immediately. The thymus and spleen indices were calculated according to the following formula:

Thymus or spleen index (mg/g)=(weight of thymus or spleen)/body weight

### 3.6. Activities of SOD and GSH-Px

Activities of SOD and GSH-Px in liver tissue were measured using commercial kits (Nanjing Jiancheng Bioengineering Institute (NJBI), Nanjing, China).

### 3.7. Determination of Lipid Peroxidation and Reduced GSH Content in Liver Tissue

Lipid peroxidation was evaluated as MDA equivalents as described by Ohkawa *et al.* [31]. MDA content in mouse liver was assayed by the measurement of thiobarbituric acid-reactive substance (TBARS) levels spectrophotometrically at 532 nm. The results were expressed as nanomoles per milligram of protein. GSH content was measured by kits obtained from NJBI (China).

### 3.8. Statistical Analysis

All results obtained were expressed as mean ± SD. Data were analyzed statistically by one-way analysis of variance (ANOVA), using SPSS Statistical program (version 17.0; SPSS Inc.: Chicago, IL, USA). A value of *p* < 0.05 was considered as statistically significant.

## 4. Conclusions

The present study is the first to demonstrate the protective effects of ALC against ionizing radiation in ICR mice. Our results show that ALC administration prior to radiation can antagonize the decrease of the spleen index and thymus index caused by radiation, and can potently prevent oxidative damage induced by gamma radiation. ALC can almost totally reverse the effect of radiation on antioxidant markers but only partially the undesirable effects on thymus and spleen. The protective mechanisms of ALC may be attributed to its free radical scavenging activity and regulating the activity of antioxidant enzymes. However, further work is required to reveal the molecular mechanism of the radioprotective action of ALC.

## Figures and Tables

**Table 1:** Effect of ALC on thymus and spleen indices of mice. Data are given as means ± SD for 10 mice.

Group		Body weight (g)	Sleep weight (mg)	Sleep index (mg/g)	Thymus weight (mg)	Thymus index (mg/g)
NC		31.54 ± 0.97	153.62 ± 9.12 #	4.90 ± 0.70 #	76.66 ± 6.13 #	2.44 ± 0.33 #
RC		30.07 ± 0.88	44.19 ± 6.91 *	1.41 ± 0.28 *	21.65 ± 2.64 *	0.69 ± 0.27 *
ALC	50	31.43 ± 0.57	57.92 ± 7.98 *,#	1.84 ± 0.34 *,#	25.16 ± 3.07 *	0.79 ± 0.26 *
(mg/kg b.w.)	100	30.85 ± 0.75	64.35 ± 6.26 *,#	2.09 ± 0.33 *,#	60.31 ± 5.38 *,#	1.95 ± 0.31 *,#
+ Radiation	200	31.51 ± 0.6	73.30 ± 7.15 *,#	2.33 ± 0.43 *,#	61.45 ± 3.56 *,#	1.95 ± 0.31 *,#

* *p* < 0.05 *vs.* NC group;

# *p* < 0.05 *vs.* RC group.
